# Endovascular repair of a ruptured axillary artery during open reduction of the shoulder dislocation with humerus fracture

**DOI:** 10.1093/jscr/rjad660

**Published:** 2023-12-05

**Authors:** Hirokazu Matsushima, Tsunehiro Shintani, Hidenori Kita, Yuto Hasegawa

**Affiliations:** Department of Vascular Surgery, Shizuoka Red Cross Hospital, 8-2, Ottemachi, Aoi-Ku, Shizuoka, Shizuoka 420-0853 Shizuoka, Japan; Department of Vascular Surgery, Shizuoka Red Cross Hospital, 8-2, Ottemachi, Aoi-Ku, Shizuoka, Shizuoka 420-0853 Shizuoka, Japan; Department of Vascular Surgery, Shizuoka Red Cross Hospital, 8-2, Ottemachi, Aoi-Ku, Shizuoka, Shizuoka 420-0853 Shizuoka, Japan; Department of Vascular Surgery, Shizuoka Red Cross Hospital, 8-2, Ottemachi, Aoi-Ku, Shizuoka, Shizuoka 420-0853 Shizuoka, Japan

**Keywords:** ruptured axillary artery, open reduction of the shoulder dislocation, endovascular repair

## Abstract

Axillary artery injury secondary to shoulder dislocation with humerus fracture is rare. Rupture of the axillary artery during open reduction is extremely rare. Here, we report about a rare case of a ruptured axillary artery during an open reduction for shoulder dislocation with humerus fracture. A 58-year-old man with left shoulder pain because of a fall after alcohol consumption was diagnosed as having left shoulder dislocation with a humerus fracture. He underwent open reduction surgery. During the procedure, bleeding was observed, and further examination through angiography revealed an ruptured axillary artery. To address this urgent situation, stent grafts were promptly deployed retrogradely from the brachial artery. The postoperative course was uneventful, except for brachial plexus palsy. In the emergent setting, endovascular repair is an efficient alternative to conventional open surgery for controlling bleeding when a ruptured axillary artery occur during open reduction for shoulder dislocation.

## Introduction

Traumatic axillary artery injury caused by shoulder dislocation with humerus fracture is rare [1, 2]. Traditionally, open surgery (arterial suture or bypass grafting) has been the primary treatment for traumatic axillary artery injury. However, recent reports have highlighted the good prognostic course after endovascular repair. Herein, we report a rare case of a ruptured axillary artery during an open reduction for shoulder dislocation with humerus fracture. The patient was successfully treated with endovascular revascularization.

## Case report

**Figure 1:**
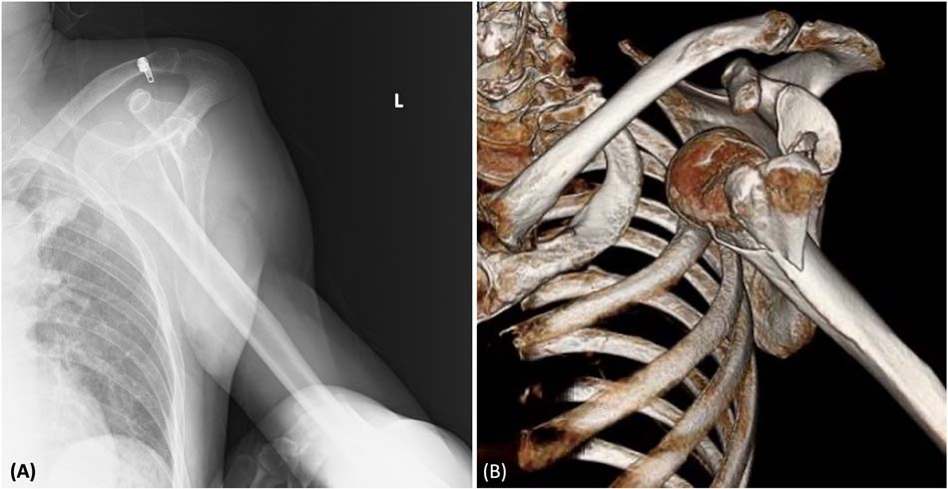
(A) Radiography showing the dislocation of the left shoulder joint. (B) 3D-CT showing a humeral fracture.

A 58-year-old man, a smoker with a history of lobectomy for lung cancer, presented to our emergency centre with complaints of left shoulder pain after striking his left shoulder in a fall after alcohol consumption. On arrival at the hospital, his blood pressure was 107/69 mmHg and his pulse rate was 78 beats/min. There was no cyanosis in his extremities. The radial artery pulse was not checked during the initial examination. Blood test results revealed a haemoglobin level of 13.2 g/dL. Radiography and computed tomography (CT) showed an anterior dislocation of the left shoulder joint with a humerus fracture (Fig. 1). The orthopaedic team did not suspect vascular injury based on physical examination and blood test, and did not perform a contrast enhanced CT. Their attempts to perform closed reduction for the shoulder dislocation were unsuccessful. Therefore, open reduction was performed under general anaesthesia.

**Figure 2:**
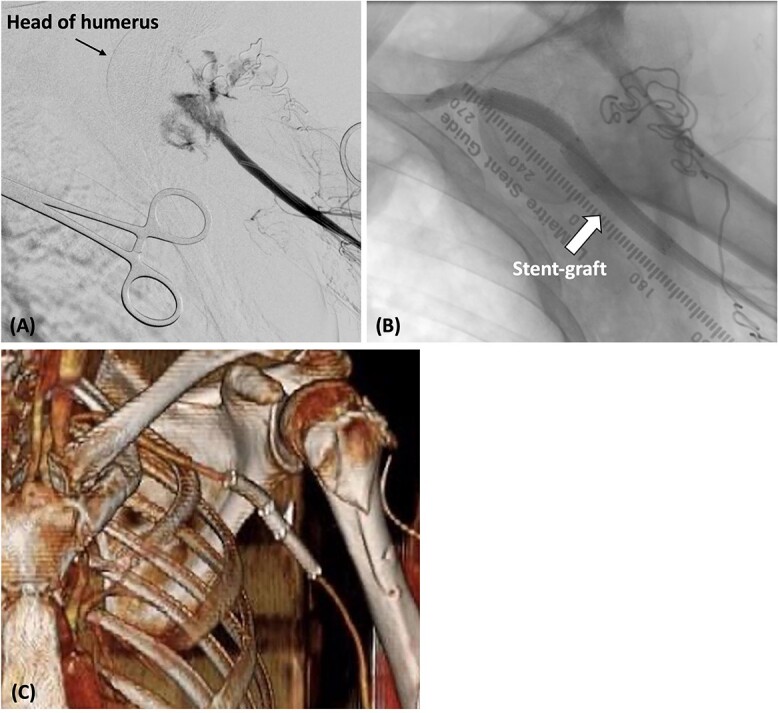
(A) The catheter was inserted from the left brachial artery, and angiography revealed the extravasation of the axillary artery. (B) Stent grafts were placed at the site of the axillary artery rupture to confirm that there was no leakage. (C) Postoperative contrast-enhanced CT revealed that blood flow in the axillary artery was maintained.

During open reduction, active arterial bleeding was identified from the incision site along with an absent left radial artery pulse. After consultation, we exposed the left brachial artery and inserted a 6-Fr sheath. Angiography revealed a rupture of the axillary artery (Fig. 2A). Because we were unable to penetrate the injured axillary artery with the guidewire from the brachial artery, we placed a 4-Fr catheter (Judkins Right4.0, MEDIKIT CO., Ltd., Tokyo, Japan) at the origin of the left subclavian artery from a right femoral approach. We then advanced a 0.014-inch guidewire (Jupiter FC Guide Wire, Boston Scientific, Marlborough, MA, USA) along a 1.9-Fr/2.5-Fr Fencer microcatheter to the left brachial artery. A 6-Fr snare catheter was inserted through the left brachial artery. The guidewire inserted from the right femoral artery was pulled through the left brachial artery. Intravascular ultrasonography was used to measure the inner diameter of the artery on the proximal and distal sides of the axillary artery injury site (7.7 mm on the proximal side and 6.5 mm on the distal side). Two Gore Viabahn stent grafts (7 × 50 mm and 8 × 50 mm; W. L. Gore and Associates Inc., Flagstaff, AZ, USA) were deployed retrogradely from the brachial artery (Fig. 2B). The stent grafts were expanded using an 8.0 × 60-mm balloon (SHIDEN HP: Kaneka Medical Products, Japan). Final angiography showed good blood flow inside the stent graft with no endoleaks. The reduction of the shoulder dislocation was then completed.

Postoperatively, cefazolin was administered intravenously for 1 week after endovascular repair. The radial artery was palpable, and contrast-enhanced CT confirmed that the absence of any leakage from the axillary artery (Fig. 2C). The patient experienced a smooth recovery, except for the presence of brachial plexus palsy. He was discharged on the 10th day, and no infection of the wound or stent grafts was observed during the 5-month outpatient follow-up.

## Discussion

Axillary artery injuries occur in ~1% of shoulder dislocations [1, 2]. The third part of the axillary artery is the vulnerable segment in these cases as it is anchored by the circumflex humeral and subscapular arteries [2].

Several mechanisms have been postulated to explain the rupture of the axillary artery in our case. First, it is hypothesized that the intimal layer of the axillary artery has less elasticity than the adventitia, and traction during trauma could have caused the intimal layer to tear, resulting in axillary artery dissection [3]. Unfortunately, contrast-enhanced CT was not performed before the procedure in our case. Therefore, it is unclear whether artery dissection had occurred before the procedure. Second, a humerus fracture also occurred in our case, and the axillary artery located at the fracture site was ruptured. This suggests that the fracture fragment may have exacerbated the axillary artery injury. Third, it is unclear whether the axillary artery injury occurred at the time of the shoulder dislocation or during the manipulation of the open reduction. We suspect that the manipulation during the reduction may have ruptured the fragile arterial wall as arterial haemorrhage occurred immediately after humeral rotation during the open reduction.

Our case was an older male patient with risk factors for atherosclerosis, such as smoking and lung cancer. It has been reported that rupture of the axillary artery by traction because of blunt trauma is more likely to occur in older patients or patients with atherosclerotic changes in the vessels [2]. The mortality rate of traumatic axillary artery injury has been reported to be as high as 10% [4]. In our case, the orthopaedic team did not suspect vascular injury, so they did not confirm the radial artery pulse or perform a vascular evaluation of axially artery using contrast enhanced CT before open reduction. However, older adults with severe shoulder dislocation should undergo a vascular evaluation of the axillary artery.

Traditionally, traumatic axillary artery injuries have been treated with open surgery, such as arterial sutures or bypass grafting. However, favourable outcomes of endovascular treatment in blunt and penetrating trauma have recently been reported [5]. The advantages of endovascular treatment include avoiding surgical dissection and damage to adjacent structures in bleeding situations, reducing blood loss, and shortening the hospital stay. However, the risk of infection, the durability of stent grafts, and stent graft deformation in highly mobile anatomical regions remain challenging. Boggs *et al*. [5] reported no early postoperative stent graft deployment failures, stent graft migration, thrombosis, or surgical site infection in their study. If an axillary artery injury was known or highly suspected preoperatively, preparing the proximal axillary artery for potential active bleeding and opting for open surgery would be the first choice. In contrast, endovascular repair can be advantageous when urgent control of bleeding from a ruptured axillary artery is necessary, as in our case where axillary artery injury was not suspected preoperatively. However, the long-term safety of stent grafts is unclear, and long-term follow-up is required.

In conclusion, axillary artery injury should always be considered in patients with severe shoulder dislocation, and confirmation of the radial artery pulse and contrast enhanced CT should be performed for vascular evaluation. However, in the emergent setting such as a ruptured axillary artery, endovascular treatment would be a viable alternative to traditional open surgery for control of active bleeding.
